# Impact of a postcrash first aid educational program on knowledge, perceived skills confidence, and skills utilization among traffic police officers: a single-arm before-after intervention study

**DOI:** 10.1186/s12873-020-00317-y

**Published:** 2020-03-18

**Authors:** Menti L. Ndile, Gift G. Lukumay, Karin Bolenius, Anne H. Outwater, Britt-Inger Saveman, Susann Backteman-Erlanson

**Affiliations:** 1 Department of Clinical Nursing, Muhimbili University of Health and Allied Sciences (MUHAS), P.O BOX 65001, Dar es Salaam, Tanzania; 2 Department of Community Nursing, MUHAS, Dar es Salaam, Tanzania; 3 Department of Nursing, Umeå University, Umeå, Sweden

**Keywords:** Traffic police, Postcrash, First aid education, Road injuries

## Abstract

**Background:**

An overwhelming proportion of road traffic deaths and injuries in low- and middle-income countries (LMICs) occur in prehospital environments. Lay first responders such as police officers play an important role in providing initial assistance to victims of road traffic injuries either alone or in collaboration with others. The present study evaluated a postcrash first aid (PFA) educational program developed for police officers in Tanzania.

**Method:**

A 16-h PFA educational program was conducted in Dar es Salaam, Tanzania, for 135 police officers. Participants completed training surveys before, immediately and 6 months after the training (before, *N* = 135; immediately after, *N* = 135; after 6 months, *N* = 102). The primary outcome measures were PFA knowledge, perceived skills confidence, and skills utilization. Parametric and nonparametric tests were used to analyse changes in outcome.

**Results:**

The mean PFA knowledge score increased from 44.73% before training (*SD* = 20.70) to 72.92% 6 months after training (*SD* = 18.12), *p* < .001, *N* = 102. The mean PFA perceived skills confidence score (measured on a 1–5 Likert scale) increased from 1.96 before training (*SD* = 0.74) to 3.78 6 months after training (*SD* = 0.70), *p* < .001, *N* = 102. Following training, application of the recovery position skill (*n* = 42, 46%) and application of the bleeding control skill (*n* = 45, 49%) were reported by nearly half of the responding officers. Less than a quarter of officers reported applying head and neck immobilization skills (*n* = 20, 22%) following training.

**Conclusion:**

A PFA educational program has shown to improve police officers’ knowledge and perceived skills confidence on provision of first aid. However qualitative research need to be conducted to shed more light regarding reasons for low utilization of trained first aid skills during follow-up.

## Background

More than 1.2 million people worldwide die every year as a result of injuries related to road traffic crashes, and as many as 50 million people continue to experience suffering as a result of such injuries [1]. More than half of all deaths resulting from road traffic injuries (RTIs) occur among vulnerable road users such as pedestrians, cyclists, and motorcyclists. Also, an overwhelming proportion of these deaths occur in prehospital environments in low- and middle-income countries (LMICs) [2]. Inadequate emergency transport services and a lack of qualified health-care providers are among the main obstacles to effective emergency prehospital care. For instance, in more than half of African countries, less than 10% of seriously injured patients benefit from ambulance evacuation [3]. Most RTI victims get to the hospital from the scene through the efforts of untrained civilians and medically unknowledgeable lay responders such as police officers [4–7].

The World Health Organization (WHO) recommends that where no prehospital trauma care system exists, first responder care should be established through the involvement of members of the community [8]. Based on the WHO recommendation, several educational programs have been piloted across Africa to build knowledge and skills capacity of lay first responders to recognize an emergency, call for help, and provide initial care until formally trained health-care personnel can take responsibility [9–12]. Previous studies of educational programs have focused on drivers or a mix of participants. Apart from being small studies, most of them did not go further to assess the application of trained knowledge and skills in the work environment. The present study not only focused on an educational program for police officers, but also assessed the application of the trained skills in the work environment.

In Tanzania, traffic police officers are officially responsible for providing first aid and for facilitating the transport of injured people from the crash scene to the hospital, either independently or in collaboration with other lay responders. Although a first aid course is part of the curriculum during police officers’ formal trainings, a previous study conducted to investigate their knowledge, practice, and attitudes toward care of RTI victims revealed that they have low levels of knowledge and poor practices [13]. Some of the reasons for this finding could be attributed to the fact that the curriculum and training approach to the management of road trauma victims is inadequate.

The purpose of postcrash first aid (PFA) educational programs is to provide traffic police officers with updated trauma care knowledge and skills by means of evidence-based teaching methods to ensure the effective transfer of knowledge and skills.

In light of this, two research questions were addressed in the present study: (1) What were the levels of knowledge and perceived skills confidence among traffic police officers before and after a PFA educational program? (2) What was the level of skills utilization among traffic police officers after a PFA educational program?

We hypothesized that first aid knowledge and perceived skills confidence among traffic police officers would improve after implementation of a PFA educational program.

## Method

### Design

A before-after single-arm pilot study involving a cohort of traffic police officers was initiated in June 2018. The study aimed to evaluate the impact of an educational program on knowledge, perceived skills confidence, and utilization of trained skills in postcrash first aid (PFA). The study protocol was registered retrospectively at the Registry for International Development Impact Evaluation with ID number: RIDIE-STUDY-ID-5bb71e0ed1e89.

### Setting

The present study was conducted in Dar es Salaam Region, Tanzania. The region, which has an area of 1590 km^2^, is the location of the city of Dar es Salaam which is a major commercial seaport and Tanzania’s largest city, with an estimated population of more than 5.7 million [14]. Dar es Salaam Region was selected as the setting for the present study because, according to a 2016 report by the Tanzania Police Force and the National Bureau of Statistics, it has the nation’s highest number of road traffic incidents, accounting for more than a third of all such incidents per year [15]. Almost all major road intersections and road crash hotspots in Dar es Salaam are lined with traffic police posts for observation of road safety. Currently there are more than 70 traffic police posts with a distance from one post to another ranging between one to one and half kilometre apart along the road. Presence of traffic police posts and communication system may facilitate availability of traffic police in the event of a crash.

### Participants

Traffic police officers were recruited to participate in the present study for three main reasons: (1) First aid provision is part of their job description; (2) they are authority figures and they command the crash scene; and (3) they are readily available at the crash scene. Details on participant background characteristics such as age, sex, educational background, and work experiences are provided in Table 1.

**Table 1:** Baseline characteristics of the study participants (*N* = 135)

Characteristics	n	%
**Sex**		
Male	88	65.2
Female	47	34.8
**Age group (years)**		
20–29	29	21.5
30–39	61	45.2
40–49	35	25.9
50–59	10	7.4
**Highest educational attainment**		
Primary school	11	8.1
Ordinary secondary school	96	71.1
Advance secondary school	7	5.2
College	16	11.9
University	5	3.7
**Work experience (years)**		
< 10	113	83.7
10–20	20	14.8
> 20	2	1.5
**Previous on-the job first aid training**		
Yes	25	18.5
No	110	81.5
**Number of RTI victims cared one year before training**		
0	31	23.0
1–5	55	40.7
6–10	15	11.1
> 10	34	25.2

*Note*. *RTI* Road Traffic Injury

### Sample size

Using the online Statistics for Psychologists program (AICBT Ltd., https://www.ai-therapy.com/psychology-statistics/terms/), we estimated that sample size should be 128 participants to detect an effect size (Cohen’s *d*) of 0.25 for trauma first aid knowledge as primary outcome, at an alpha error rate of .05 (two-tailed) and a beta error rate of .20. An additional 10% was added to account for loss to follow-up; thus, the final sample size was 141 participants. An effect size of 0.25 was chosen on the basis of empirical distributions of effect sizes from comparable educational intervention studies that considered it of practical significance [16].

### PFA program

The PFA educational program focused on imparting basic knowledge and skills to traffic police on managing injury victims at the scene and on the way to the hospital. The course program was developed in accordance with WHO guidelines on essential knowledge, skills, equipment, and supplies for the provision of basic first aid [8]. Three experienced emergency and trauma care teachers facilitated the course. Topics covered during the training included scene survey, provider safety, and initial assessment and care of the injury victim. Initially, lectures and discussions on topics were conducted in groups of about 27 participants per session. Afterward, participants were divided into groups of about 8 to 10 for practical training. Role-play and mannequins were used to impart practical skills. The course content was covered in 2 days (a total of 16 h of session time). At the end of the training, the traffic police were given leaflets on basic steps in managing injury victims for use as a reference.

### Data collection and procedures

Permission for conducting PFA training and data collection for evaluating the training was granted by the office of Inspector General of Police. The recruitment process was left under the mandate of researchers who ensured rights of officers to participate or decline participation were maintained without interference from police management. A list of 340 police officers from a previous database regarding knowledge, self-reported practice, and attitudes of traffic police officers was used to proportionately select officers from jurisdictional areas of Kinondoni, Ilala and Temeke [13]. A simple random sampling by drawing a YES/NO tickets from the box was used. 141 police officers who drew YES tickets were invited to participate in the study, whereof 135 officers attended the training and completed the before and after questionnaires. The reason for those not attending and completing the training was mainly due to sickness, attending emergencies and being assigned other tasks. Before training sessions, selected officers were informed about the purpose and duration of the educational program and then were asked for their consent to participate.

Data were collected before and immediately after the educational program by questionnaire. Six months after the training, the police officers were contacted by telephone to identify their work locations; the researchers then physically visited the identified locations and asked the officers to complete a follow-up questionnaire. An updated version of a self-administered questionnaire from a previous study was used in the present study for data collection [13].

**Fig. 1:**
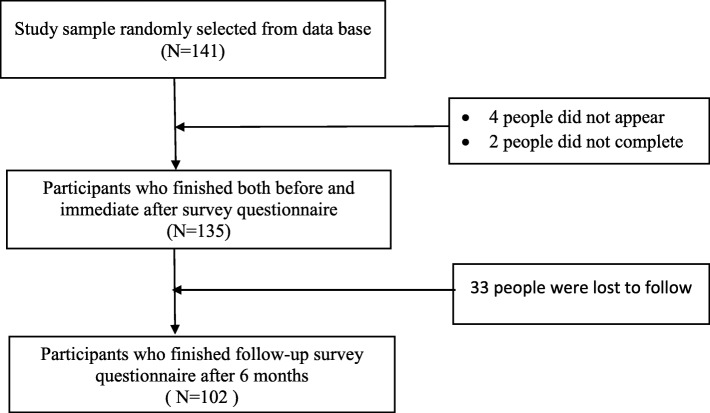
Participant recruitment and follow-up process. Flowchart Summarizing Participant Recruitment and Follow-up Processes

The process through which officers were recruited and follow-up was executed is summarized in Fig. 1.

### Questionnaire

The questionnaire was developed on the basis of WHO guidelines on essential knowledge and skills for provision of basic first aid [8]. A Swahili version already existed and was tested for reliability; the alpha value of .74 for the knowledge domain indicated acceptable reliability of the scale [13]. We modified the existing Swahili version of the questionnaire by adding more knowledge items and developed new questions on assessing perceived skills confidence, utilization of trained skills, and training experience. Two experts in emergency and trauma training in Tanzania assessed the entire questionnaire for content validity. Face validity was ensured by piloting the questionnaire to 15 traffic police officers who did not take part in the main study. Minor structural changes such as sequencing questions according to systematic trauma assessment were made. The questionnaire consisted of 34 items; baseline characteristics of participants such as sex, age, educational level, work experience, previous first aid training, and number of injured persons cared for were included. Cronbach’s alpha coefficients for reliability of the test in all domains (knowledge, perceived skills confidence, and training experience) in the questionnaire ranged from .70 to .94 when measured before, immediately after, and 6 months after training. The values indicate that the items within each domain were consistent in measuring the same attributes [17]. Details of respective outcome measures in the questionnaire are described below.

### Outcome measures

Three primary outcomes were measured: knowledge of PFA, perceived PFA skills confidence, and PFA skills utilization.

#### Knowledge of PFA

The same eight multiple choice questions assessing participants’ knowledge of PFA were asked before, immediately after, and 6 months after PFA training. Questions involved condition given priority to care in case of multiple injuries, assessment of consciousness, airway management, breathing assessment, control of external bleeding, care of fracture of extremities, positioning of victim, and head and neck immobilization. For each knowledge question, a correct response was scored one and an incorrect response was scored zero. The item scores were summed up and divided by the number of items to get an average score, which was then converted into a percentage score.

#### Perceived PFA skills confidence

The same six questions assessing participants’ perceived PFA skills confidence were asked before, immediately after, and 6 months after PFA training. The questions were about management of compromised airway, breathing assessment, control of external bleeding, care of fractures of extremities, positioning victim, and head and neck immobilization. Questions were answered on a 5-point (1 = very low, 2 = low, 3 = moderate, 4 = high, and 5 = very high). Item scores were summed up and divided by the number of items to get an average score.

#### Utilization of trained PFA skills

Six questions on managing compromised airway, breathing assessment, control of external bleeding, care of fracture of extremities, positioning victim, and head and neck immobilization were answered on a 4-point Likert scale 6 months after the intervention (1 = never, 2 = sometimes, 3 = often, 4 = always). Percentages were calculated to determine utilization of skills based on Likert scale categories.

One secondary outcome, *PFA training experience*, was measured. Five questions on overall PFA training experience were answered on a 4-point Likert scale (1 = strongly disagree, 2 = disagree, 3 = agree, 4 = strongly agree).

Respective Likert scales are common in measuring participants’ responses in different contexts. They were chosen based on literature review [18]. For details on overall questions, see Additional file 1: PFA questionnaire.

### Data analysis

To perform data analysis, we used the Statistical Package for Social Sciences (SPSS), version 24. Demographic characteristics were reported as means, standard deviations, counts, and percentages.

Paired *t* tests were used to compare differences in participants’ PFA knowledge scores before training (T0), immediately after (T1), and 6 months following completion of training (T2). A repeated-measures analysis of variance (RM-ANOVA) was used to compare differences in scores within subjects using the measurement points (T0, T1 and T2). A Bonferroni adjustment was made for multiple comparisons to control for familywise error rates; with this correction, we set *p* < .017 as our significance level [19]. We used McNemar’s test to compare individual PFA knowledge items scores (correct and incorrect responses) and the measurement points (T0, T1 and T2) for association.

Regarding perceived PFA skills confidence, since data from Likert scales is ordinal and tends to be non-normally distributed, a nonparametric test (Wilcoxon sign rank test) should ideally be conducted and reported. Considering that in the present study the nonparametric test and the parametric test (paired *t* test) provided almost the same conclusion about the change in perceived skills confidence, we report the means, which present the results better than the medians [20]. Spearman rank-correlation analysis was used for the assessment of the interrelationships among outcome measures and baseline characteristics such as age, sex, educational level, and work experience.

## Results

### Baseline characteristics

Traffic police officers (*N* = 135) completed a training questionnaire before and immediately after training. About two thirds of the participants were male (*n* = 88; 65%) and two thirds were in the 30–39 years age interval (*n* = 61; 45%). The mean age was 36.25 years (*SD* = ± 8.0). More than two thirds of the participants (*n* = 96; 71%) had completed their education at the ordinary secondary school level. Regarding work experience, more than three quarters (*n* = 113; 84%) had work experience of less than 10 years in the traffic police department. More than three quarters of the officers (*n* = 110; 81%) had not received on-the-job first aid training other than what they had received during police training. Among officers who reported providing care to RTI victims, more than three quarters (*n* = 104; 77%) said they had cared for at least one RTI victim in the previous year. (Table 1 provides additional details on baseline information).

### Follow-up information

A total of 102 traffic police officers completed the follow-up questionnaire while 33 did not (response rate 76%). There was no significant difference in background characteristics between those who completed follow-up questionnaire and those who did not. The reasons for not completing the follow-up questionnaire were mainly due to relocation to other regions, maternity leave, long vacation, being in studies, and long sickness. The officers who completed the follow up questionnaire (*n* = 88; 86%) said they provided care at least to one RTI victim in the 6 months after the intervention. Thirty officers said they provided care to more than 10 RTI victims during the 6 months after the intervention.

### PFA knowledge

The overall mean PFA knowledge score (*N* = 135) increased from 44.44% before training (*SD* = 21.20) to 84.54% immediately after training (*SD* = 13.76), *p* = < .001. It increased (*N* = 102) from 44.73% before training (*SD* = 20.70) to 72.92% 6 months after training (*SD* = 18.12), *p* = < .001. The results indicated a significant time effect on scores when PFA knowledge was measured before, immediately after, and 6 months after training (*N* = 102 pairs), *p* = < .001.

Immediately after training, statistically significant improvements were observed on all PFA knowledge score items when compared to the before-training condition. At 6 months post-training, statistically significant improvements on PFA knowledge score items were observed in all areas, with the exceptions of prioritizing care and assessing breathing, relative to the before-training condition; however, for these two items, scores were slightly higher compared to the before-training condition (see Table 2).

**Table 2:** Correct knowledge scores before, immediately after and after six months following training (*N* = 102)

	Before training	Immediately after training	Six months after training		
	T0	T1	T2	T0-T1	T0-T2
Knowledge items	n (%)	n (%)	n (%)	*p* value	p value
Prioritizing care	90 (88.2)	102 (100)	98 (96.1)	NA	0.077
Assess responsiveness	53 (52)	97 (95.1)	76 (74.5)	0.001*	0.001
Open airway	13 (12.7)	56 (54.9)	52 (51)	0.001*	0.001*
Assess breathing	50 (49)	87 (85.3)	61 (59.8)	0.001*	0.135
Control external	45 (44.1)	90 (88.2)	70 (68.6)	0.001*	0.001
Care of fractures	64 (62.7)	87 (85.3)	84 (82.4)	0.001*	0.001
Recovery position	25 (24.5)	91 (89.2)	83 (81.4)	0.001*	0.001*
Head & neck immobilization	25 (24.5)	69 (67.6)	71 (69.6)	0.001*	0.001*

*P< 0.001, statistically significant at *P*< 0.05 (McNemar’s test)

T0 = Before training, T1 = Immediately after training, T2 = After six months following training

*NA* = No statistics computed because T1 is constant

### Perceived PFA skills confidence

The mean PFA skills confidence score (*N* = 135) increased from 1.98 before training (*SD* = 0.76) to 4.15 immediately after training (*SD* = 0.22), *p* < .001. The mean PFA skills confidence score (*N* = 102) increased from 1.96 before training (*SD* = 0.74) to 3.78 6 months after training (*SD* = 0.70), *p* < .001.

Immediately after training, statistically significant improvements in perceived PFA skills confidence scores were observed on all skills items relative to the before-training condition. At 6 months post training, statistically significant improvement in perceived PFA skills confidence scores were also observed on all skill items (see Table 3).

**Table 3:** Mean Perceived PFA Skills Confidence Scores on Respective First Aid Procedures (1 = very low, 5 = very high, *N* = 102)

	Before training	Immediately after training	Six months after training	T0-T1	T0-T2
Skill items	(T0) mean (SD)	(T1) mean (SD)	(T2) mean (SD)	*p value	* *p* value
Open airway	1.53 (0.84)	4.56 (0.70)	3.80 (0.83)	0 .001	0.001
Assess breathing	1.91 (1.0)	4.70 (0.48)	3.76 (0.94)	0 .001	0.001
Control external bleeding	2.42 (1.37)	3.95 (0.22)	3.75 (0.81)	0 .001	0.001
Care of fractures	2.54 (1.33)	3.96 (0.30)	3.82 (0.80)	0 .001	0.001
Recovery position	1.85 (1.20)	3.95 (0.22)	3.80 (0.78)	0.001	0.001
Head & neck immobilization	1.51 (0.91)	3.87 (0.41)	3.75 (1.0)	0 .001	0.001

*P< 0.001, statistically significant at *P*< 0.05

### Skills utilization

**Fig. 2:**
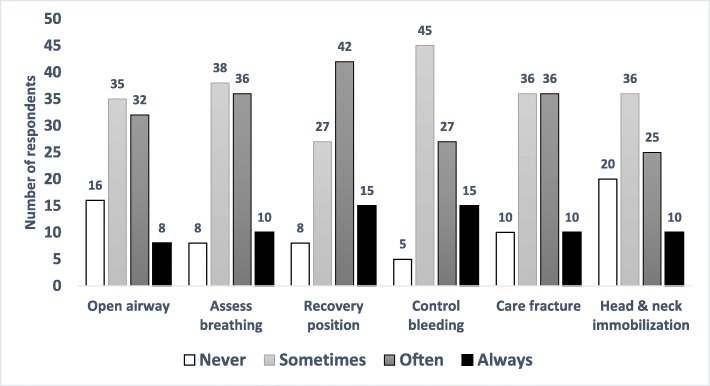
Post Intervention First Aid Skills Utilization. Number of Participants Who Reported Using the Individual First Aid Skills, Six Months Post-Training

Six months after the completion of training, 10 participants reported that they had never applied any of the trained skills because they had not encountered any RTI victims during that time; 92 participants reported that they had had an opportunity to apply the skills. Of that group of 92 officers, nearly half (*n* = 42, 46%) reported that recovery position skills were often applied. About half of the police officers (*n* = 45, 49%) reported that they had sometimes applied bleeding control skills. In contrast, only about a quarter of the officers (*n* = 20, 22%) reported having applied head and neck immobilization skills. Further details are provided in Fig. 2.

### Correlation between outcome measures and baseline characteristics

With one exception, application of the Spearman rank test indicated that there was no correlation between knowledge scores, perceived skills confidence scores, and skills utilization at any of the three study phases (before, immediately after, and after 6 months) when a comparison was made to baseline characteristics; the exception was a positive correlation between educational level and knowledge scores before intervention (*r* = .218).

## Discussion

The critical resources and infrastructure needed to provide prehospital emergency care—dedicated transport, human resources, and facilities to manage road trauma are scarce in Tanzania. Recently, the government has initiated strategies to improve the provision of emergency care services. Central to this effort is the development of the Emergency Medical Service (EMS) guideline. The guideline aims to ensure a standardized approach to prehospital patient care in the country. One important element in the provisional guideline is building the capacity of laypersons to provide emergency prehospital care. This can be achieved by conducting evidence-based training relevant to the level of care and context. In line with the EMS guideline, the aim of the present study was to evaluate the impact of PFA educational training on the first aid knowledge and skills confidence of traffic police officers.

We tested the hypothesis that a PFA educational program delivered to traffic police would lead to improved knowledge and increased confidence in first aid delivery skills. Before the training, police officers had low levels of knowledge and confidence in regard to administering first aid to RTI victims, a finding that is reflected in those of a previous survey in Tanzania [13]. Post-training surveys immediately after training and after 6 months showed that there was a significant improvement in first aid knowledge and skills confidence among traffic police. Similar studies on lay responder first aid training with participants other than police officers also demonstrated the positive impact of educational training on first aid knowledge and skills [9, 10, 21–23]. Common to these studies was the use of didactic and hands-on approaches to training. In addition, training course content covered essential basic first aid interventions as recommended by WHO for use in resource-limited settings [8]. Results from the present study and others indicate reliability in training approach to bring about positive outcomes.

Retention of trained knowledge among police officers was observed to be significant 6 months after the completion of training (*n* = 102, 72.9%). This finding corresponds to that of a similar study conducted in Uganda which showed significant retention of first aid knowledge among police officers [10].

Six months after the completion of training, about 90% of officers in the present study had used at least one trained first aid skill, the most frequently applied skills being positioning victims in the recovery position (46%), caring for fractures (39%), and conducting breathing assessments (39%). These findings closely resemble those of the study done in Uganda [10] (though that study included also participants who were not police officers) with regard to the use of the recovery position (57% in the Uganda study) and stabilization of fractures (35% in the Uganda study). In contrast, bleeding control was applied much less often in the present study (29%) relative to the Ugandan study (74%). The wide difference in applying bleeding control measures may be attributed to, among other things, a lack of availability of first aid kits and supplies such as gloves, given that such supplies were not provided after completion of the training described in the present study. It can be assumed that participants were afraid to apply the skill without protective gears for fear of being infected by any blood-borne disease.

Regarding training experience, both immediately after and 6 months after completion of the training more than 80% of traffic police in the present study reported that they had had enough time to train, that the content of the training and the teaching methods were relevant, and believed that they were well prepared to provide first aid. However this finding need to be cautiously considered as it may as well be influenced by social desirability bias.

In the context of police officers’ work environment, factors such as a lack of first aid supplies, other responsibilities, and the level of support from senior officers and other stakeholders may influence care provision behaviour. These factors were reported as barriers in a systematic review of the effectiveness of nonresuscitative first aid training of laypersons; the review showed that traditional first aid training is more likely to improve trainees’ competence levels than willingness to help during emergencies [24]. However, considering that 90% of police officers in the present study used at least one of their trained skills, it would be interesting to learn the motivation behind this despite challenging work conditions.

### Limitations of the study

The present study had several limitations. First, it did not have a control group, and thus it cannot be stated conclusively that the observed change in knowledge and perceived skills confidence was caused by the training program alone. However, having a control group among police working in the same jurisdictional area would have created a high risk of contamination because police officers in a potential control group would have received a lot of information regarding the course while working with police officers in the intervention group. Second, in this study, we did not provide first aid kits and materials such as gloves for police officers to use when they encountered RTI victims. Therefore, application of the knowledge acquired in training may have been affected by a lack of supplies. Third, self-reporting was used to measure one of the outcomes; in such a situation, social desirability can be a reason for a change. However, respondents were informed about the importance of being truthful and expressing their views freely, as they were assured that confidentiality would be respected.

### Suggestions for future research

Additional research is required to evaluate the impact of the first aid educational program on actual skills change; findings based on reported changes in skills, while useful, are not entirely sufficient. Furthermore, research to evaluate the impact of contextual factors on helping behaviour is required to shed more light on police officers’ willingness to help and to understand why certain skills are used more than others. Further studies involving randomised control trials are need to determine if first aid care provided is appropriate and the injury managed correctly. Cost-effectiveness analysis studies are also required to determine the affordability of the first aid intervention in the setting.

### Implications for Prehospital care practice

The educational program was found to have a significant positive impact on PFA knowledge and perceived skills confidence among police officers. Furthermore, the majority of the trainees reported using at least one trained skill to care for RTI victims after completion of the training. Finding from this study may be transferable to similar contexts and applicable in other resource limited settings where traffic police officers also work as first responders like in Tanzania.

## Conclusion

A PFA educational program has shown to improve police officers’ knowledge and perceived skills confidence on provision of first aid. However qualitative research need to be conducted to shed more light regarding reasons for low utilization of trained first aid skills during follow-up.

## Supplementary information

**Additional file 1.**

## Data Availability

The data set generated and/or analysed during the present study is not publicly available due to the confidential nature of the data, but is available from the corresponding author on reasonable request.

## Abbreviations

EMS: Emergency medical service
LMICs: Low- and middle-income countries
PFA: Postcrash first aid
RTI: Road traffic injury
WHO: World Health Organization
