# Supplementary material for: Impact of a postcrash first aid educational program on knowledge, perceived skills confidence, and skills utilization among traffic police officers: a single-arm before-after intervention study
Source: BMC Emerg Med. 2020 Mar 18;20:21. doi: 10.1186/s12873-020-00317-y (PMC7079460; doi:10.1186/s12873-020-00317-y)
Supplement: Supplementary file 1 — Additional file 1. [file 12873_2020_317_MOESM1_ESM.pdf]

MUHIMBILI UNIVERSITY OF HEALTH AND ALLIED SCIENCES

DEPARTMENT OF COMMUNITY NURSING

Survey on Impact of a Postcrash First Aid Educational Program on Knowledge, Perceived Skills Confidence, and Skills Utilization Among Traffic Police Officers

PRETEST/IMMEDIATE POST TEST QUESTIONNAIRE

We would like to invite you to participate in a survey. The aim is to know your level of knowledge and skills confidence before and immediately. Please spare with us some time to answer questions in the questionnaire. In this survey your personal information will be kept confidential. Information collected will only be used for analysis and for training purpose. Thank you!

ID Number…………………………………

PART A: Demographic data

Please TICK in the box or fill in the space were appropriate

1. Place of working
    1. Ilala [ ] 2. Kinondoni [ ] 3. Temeke [ ]
2. Age in years.....................
3. Sex
    1. Male [ ] 2. Female [ ]
4. Years of Working as traffic police officer …………….
5. Education level
    1. Primary school education [ ]
    2. Ordinary secondary school education [ ]
    3. Advanced school secondary education [ ]
    4. College (certificate, diploma) [ ]
    5. University [ ]
6. Have you ever attended road trauma first aid course? If no go to question 9
    1. Yes [ ]
    2. No [ ]
7. If yes where did you attend
    1. During police training [ ]
    2. On workshops [ ]
    3. Others mention…………………………
8. Have you ever cared RTI victim? If no go to question 10
    1. Yes [ ]
    2. No [ ]
9. If yes who many times?
    1. 1-2 times [ ]
    2. 3-6 times [ ]
    3. More than 6 times [ ]

PART B: Postcrash first aid knowledge

Please CIRCLE one correct answer from question 10 to 17

10. What is a priority problem to manage in an unconscious casualty given all problems exist on the same occassion?

    1. Bleeding
    2. Airway
    3. Fracture
    4. Burns

11. How do you check for a response to determine whether someone is unconscious?

    1. Ask questioning like ‘Can you hear me?’ and ‘Can you squeeze my hand?’
    2. Whisper in their ear and assess response
    3. Slap the casualty’s cheeks to try to get a response
    4. Shake them firmly and vigorously and assess response

12. How should you open the airway of an unconscious casualty?

    1. Head tilt and chin lift.
    2. Jaw thrust.
    3. Head tilt and jaw thrust.
    4. Lift the chin

13. How do you check for breathing of a casualty?

    1. Count the number of breaths of the casualty
    2. Check the pulse of the casualty
    3. Look, listen and feel for breaths of the casualty
    4. Check the body temperature of the casualty

14. How can you stop external bleeding?

    1. Apply tourniquets above the wound
    2. By applying direct pressure over the wound
    3. Put some amount of salt to the wound
    4. Apply an adhesive strip to the wound

15. If you suspect fracture of the leg you would

1. Support the leg using splint
2. Push the bone back in place
3. Place the leg in proper alignment
4. Wrap the area tightly with cloths

16. Which position will you place a casualty who is unresponsive and has difficulty breathing

1. Keep them on their back and raise their legs
2. Place casualty on his/her side, and keep the airway open
3. Leave them exactly where you found them
4. Gently roll the casualty onto his/her stomach with their head to one side

17. If you suspect cervical spinal injury, three consecutive actions you can take to protect the neck of a casualty when providing first aid are.

A. Keep the casualty still – tell the casualty not to move
B. Put rolled up clothing on each side of the casualty's head to keep it from moving.
C. Kneel behind the head and place your hands on either side to support it – with the head, neck and spine in a straight line

1: A, B, C
2: A, C, B
3: C, B, A
4: B, A, C

PART C: Perceived skills confidence

Circle the appropriate number to indicate your degree of skills confidence in managing road crash casualty in the listed topics **BEFORE** the training and **AFTER** the training

**Skills**	**Before training**					**After training**				
	**Low**				**High**	**Low**				**High**
18. Open compromised airway	1	2	3	4	5	1	2	3	4	5
19. Check for presence of breathing	1	2	3	4	5	1	2	3	4	5
20. Place casualty in recovery position	1	2	3	4	5	1	2	3	4	5
21. Control external bleeding	1	2	3	4	5	1	2	3	4	5
22. Managing fractures of extremity	1	2	3	4	5	1	2	3	4	5
23. Head and neck immobilization	1	2	3	4	5	1	2	3	4	5

PART D: Training experience (Applicable after completion of training**)**

To what extent do you agree or disagree with the following statements about your overall first aid training experience.

**Statement**	**Strongly disagree**	**Disagree**	**Agree**	**Strongly agree**
24. I had enough time to practice skills	1	2	3	4
25. The participant materials helped me learn the skills.	1	2	3	4
26. The teaching method helped me learn the skills.	1	2	3	4
27. As a result of the course, I feel better prepared to respond to an emergency	1	2	3	4
28. I would recommend this course to others.	1	2	3	4

END

POST TEST SIX MONTHS AFTER TRAINING

Background information:

We would like to invite you to participate in a second survey after you have received training. The aim is to evaluate the impact of first aid course you attended. Please spare with us some time to answer questions in the questionnaire. In this survey your personal information will be kept confidential. Information collected will only be used for analysis and for training purpose. Thank you!

ID Number……………………………………

PART A: Demographic data

Please TICK in the box or fill in the space were appropriate

1. Place of working

2. Ilala ☐ 2. Kinondoni ☐ 3. Temeke ☐

2. Age in years.....................

3. Education level

1. Primary school education ☐
2. Ordinary secondary school education ☐
3. Advanced school secondary education ☐
4. College (certificate, diploma) ☐
5. University ☐

4. How many road traffic injured victims have you cared/ assisted in the last six months?

1. None ☐
2. 1-5 ☐
3. 6-10 ☐
4. Above 10 ☐

PART B: Postcrash first aid knowledge

Please CIRCLE one correct answer from question 5 to 12

5. What is a priority problem to manage in an unconscious casualty given all problems exist on the same occasion?
    1. Bleeding
    2. Airway
    3. Fracture
    4. Burns

6. How do you check for a response to determine whether someone is unconscious?
    1. Ask questioning like ‘Can you hear me?’ and ‘Can you squeeze my hand?’
    2. Whisper in their ear and assess response
    3. Slap the casualty’s cheeks to try to get a response
    4. Shake them firmly and vigorously and assess response

7. How should you open the airway of an unconscious casualty?
    1. Head tilt and chin lift.
    2. Jaw thrust.
    3. Head tilt and jaw thrust.
    4. Lift the chin

8. How do you check for breathing of a casualty?
    1. Count the number of breaths of the casualty
    2. Check the pulse of the casualty
    3. Look, listen and feel for breaths of the casualty
    4. Check the body temperature of the casualty

9. How can you stop external bleeding?
    1. Apply tourniquets above the wound
    2. By applying direct pressure over the wound
    3. Put some amount of salt to the wound
    4. Apply an adhesive strip to the wound

10. If you suspect fracture of the leg you would
   1. Support the leg using splint
   2. Push the bone back in place
   3. Place the leg in proper alignment
   4. Wrap the area tightly with cloths

11. Which position will you place a casualty who is unresponsive and has difficulty breathing
   1. Keep them on their back and raise their legs
   2. Place casualty on his/her side, and keep the airway open
   3. Leave them exactly where you found them
   4. Gently roll the casualty onto his/her stomach with their head to one side

12. If you suspect cervical spinal injury, three consecutive actions you can take to protect the neck of a casualty when providing first aid are.
   A. Keep the casualty still – tell the casualty not to move
   B. Put rolled up clothing on each side of the casualty's head to keep it from moving.
   C. Kneel behind the head and place your hands on either side to support it – with the head, neck and spine in a straight line

   1: A, B, C
   2: A, C, B
   3: C, B, A
   4: B, A, C

PART C: Perceived skills confidence

Circle the appropriate number to indicate your degree of skills confidence in managing road crash casualty **AFTER** attending first aid training 6 months ago

**Skills**	**After training**
	**Low** **High**
13. Open compromised airway	1 2 3 4 5
14. Check for presence of breathing	1 2 3 4 5
15. Place casualty in recovery position	1 2 3 4 5
16. Control external bleeding	1 2 3 4 5
17. Managing fractures of extremity	1 2 3 4 5
18. Head and neck immobilization	1 2 3 4 5

PART D: Utilization of skills

After participation in the First Aid program how frequently have you applied the skills in your job? Circle the appropriate number to indicate the degree of use.

**Skills**	**Never**	**Sometimes**	**Often**	**Always**	**NA**
19. Open compromised airway	1	2	3	4	5
20. Check for presence of breathing	1	2	3	4	5
21. Place casualty in recovery position	1	2	3	4	5
22. Control external bleeding	1	2	3	4	5
23. Managing fractures of extremity	1	2	3	4	5
24. Head and neck immobilization	1	2	3	4	5

PART E: Training experience

Circle the appropriate number to indicate the extent to which you agree or disagree with the following statements about your overall first aid training experience.

**Statement**	**Strongly disagree**	**Disagree**	**Agree**	**Strongly agree**
25. I had enough time to practice skills	1	2	3	4
26. The participant materials helped me learn the skills.	1	2	3	4
27. The teaching method helped me learn the skills.	1	2	3	4
28. As a result of the course, I feel better prepared to respond to an emergency	1	2	3	4
29. I would recommend this course to others.	1	2	3	4

END
